# 2022年毛细管电泳技术年度回顾

**DOI:** 10.3724/SP.J.1123.2023.02003

**Published:** 2023-05-08

**Authors:** Yalun WEN, Yuchen SHAO, Xinying ZHAO, Feng QU

**Affiliations:** 1.北京理工大学生命学院,北京100081; 1. School of Life Science, Beijing Institute of Technology, Beijing 100081, China; 2.北京电子科技职业学院,北京100176; 2. Beijing Polytechnic, Beijing 100176, China

**Keywords:** 毛细管电泳, 2022, 年度回顾, capillary electrophoresis (CE), 2022, annual review

## Abstract

该文为2022年毛细管电泳(capillary electrophoresis, CE)技术年度回顾。归纳总结了以“capillary electrophoresis-mass spectrometry”或“capillary isoelectric focusing”或“micellar electrokinetic chromatography”或“capillary electrophoresis”为关键词在ISI Web of Science数据库中进行主题检索(排除“capillary electrochromatography”“microchip”“microfluidic”“capillary monolithic column”)得到的2022年CE技术相关研究论文881篇。重点介绍了影响因子(IF)大于10.0的*Lancet Global Health*, *ACS Central Science*, *Microbiome*, *Trends in Food Science & Technology*, *TrAC-Trends in Analytical Chemistry*, *Journal of Pharmaceutical Analysis*, *Journal of Cachexia*, *Sarcopenia and Muscle*, *Food Hydrocolloids*, *Science of the Total Environment*和*Carbohydrate Polymers*发表的16篇论文;IF在5.0~10.0内的代表性期刊*Analytical Chemistry*, *Analytica Chimica Acta*, *Talanta*和*Food Chemistry*的46篇论文;对IF小于5.0但CE技术报道较为集中的*Journal of Chromatography A*和*Electrophoresis*以及国内重要的中文核心期刊(北京大学)中的代表性工作进行了概述。本文根据国际通用学术水平评价指标之一的影响因子选择期刊,结合期刊发表CE论文代表性工作进行介绍,便于读者快速了解CE技术在过去一年的重要研究进展。

## Abstract (en)

This article provides a detailed review of capillary electrophoresis (CE) technology in 2022, summarizing a total of 881 CE technology-related articles searched from ISI Web of Science using the keywords “capillary electrophoresis mass spectrometry” or “capillary isoelectric focusing” or “micellar electrokinetic chromatography” or “capillary electrophoresis” (excluding “capillary electrochromatography”“microchip” “microfluidic” “capillary monolithic column”). The review focuses on 16 articles published in *Lancet Global Health*, *ACS Central Science*, *Microbiome*, *Trends in Food Science & Technology*, *TrAC-Trends in Analytical Chemistry*, *Journal of Pharmaceutical Analysis*, *Journal of Cachexia*, *Sarcopenia and Muscle*, *Food Hydrocolloids*, *Science of the Total Environment*, *and Carbohydrate Polymers* with impact factors (IFs) greater than 10.0, and 46 articles published in *Analytical Chemistry*, *Analytica Chimica Acta*, *Talanta*, and *Food Chemistry* with IFs between 5.0 and 10.0. A comprehensive overview of representative CE works published in *Journal of Chromatography A*, *Electrophoresis*, and important Chinese core journals (Peking University) with IFs<5.0 is also provided. Based on IFs, this review introduces representative works on CE to facilitate readers’ understanding of important research advances in CE technology over the last year.

截至2022年12月31日,以“capillary electrophoresis-mass spectrometry”或“capillary isoelectric focusing”或“micellar electrokinetic chromatography”或“capillary electrophoresis”为关键词在ISI Web of Science数据库中进行主题检索(排除“capillary electrochromatography”“microchip”“microfluidic”“capillary monolithic column”),检索到期刊论文共计881篇。影响因子(IF)>10.0的部分顶级期刊*Lancet Global Health*(IF=38.9), *ACS Central Science*(IF=18.7), *Microbiome*(IF=16.8), *Trends in Food Science & Technology*(IF=16.0), *TrAC-Trends in Analytical Chemistry*(IF=15.0), *Journal of Pharmaceutical Analysis*(IF=14.0), *Journal of Cachexia*, *Sarcopenia and Muscle*(IF=12.0), *Food Hydrocolloids*(IF=11.5), *Science of the Total Environment*(IF=10.8)和*Carbohydrate Polymers* (IF=10.7)中发表16篇毛细管电泳(capillary electrophoresis, CE)相关论文。10.0≥IF≥5.0的111种期刊中发表了CE相关论文271篇,其中*Analytical Chemistry*(IF=8.0)发表26篇,*Analytica Chimica Acta*(IF=6.9)发表10篇,*Talanta*(IF=6.6)发表7篇,影响力较大的食品分析期刊*Food Chemistry*(IF=9.2)发表3篇。IF<5.0的246种期刊中发表了CE相关论文594篇,其中与CE关联密切的*Journal of Chromatography A*(IF=4.6)和*Electrophoresis*(IF=3.6)分别发表35篇和40篇。以“毛细管电泳”为关键词,在中国知网数据库检索到12种中文核心期刊(北京大学)发表的CE相关论文15篇。

本文根据国际通用学术水平评价指标之一IF选择期刊,结合期刊发表CE论文代表性工作进行介绍,便于读者快速了解毛细管电泳技术在当年的重要研究进展。

## 1 IF>10.0的期刊

### 1.1 重要应用研究

#### 1.1.1 CE技术

新方法及其应用 Cui等^[1]^在*Journal of Pharmaceutical Analysis*发表了IgG抗体电荷异构体修饰的全局表征的研究结果,利用毛细管电泳等电聚焦-全柱成像检测技术(capillary isoelectric focusing-whole column imaging detection, cIEF-WCID)表征标准单克隆抗体类(monoclonal antibodies, mAbs)的等电点(pI),结果可与其他方法互相验证,只需μL级的样本量,6 min内可以测定pI,能够快速得到电荷异构体分布的高分辨视图。Smeets等^[2]^在*Journal of Cachexia*, *Sarcopenia and Muscle*报道了毛细管电泳-紫外检测(capillary electrophoresis with ultraviolet detection, CE-UV)测定白细胞三烯B4(leukotriene B4, LTB4)及其代谢物20-羟基白细胞三烯B4(20-hydroxy-leukotriene)的方法,4 min内完成检测,为LTB4抑制剂类药物效果评价提供了方法,为观察因细胞色素P450 4F3变异而致病的患者的临床特征提供了证据。

已报道方法的新应用 Li等^[3]^在*Food Hydrocolloids*报道了15个水稻品种主要淀粉分子结构的特征,研究了淀粉的链长分布与普通大米、速熟大米硬度和黏性的关系,可以助力开发更好质地的新一代速熟大米产品。Li等^[4]^在*Carbohydrate Polymers*报道了淀粉结构和水分含量对凝胶化性能的综合影响,首次发现直链淀粉含量与凝胶化温度呈抛物线关系,观察到不同水分含量下淀粉细分子结构与胶化参数之间不同的线性关系,为饼干等在有限水分含量下加工食品的研发提供重要参考。上述两篇报道都直接使用了Wu等^[5]^2014年在*Carbohydrate Polymers*发表的毛细管电泳-荧光检测法(capillary electrophoresis with laser-induced fluorescence detection, CE-LIF)测定线性葡萄糖聚合度的方法。Amariei等^[6]^在*Science of the Total Environment*上首次用手性观点证明了伊伐布雷定(ivabradine)对映体对海洋中费氏弧菌(*Vibrio fischeri*)的生态毒理学作用,定量检测直接使用了Casado等^[7]^2019年在*Journal of Chromatography A*发表的CE-UV测定费氏弧菌培养基中伊伐布雷定对映体含量的方法。

#### 1.1.2 CE-MS技术

新方法及其应用 Staessen等^[8]^在*Lancet Global Health*报道了CRIT-CoV-U前瞻性多中心队列研究(prospective multicentre cohort study)的结果,利用超滤离心结合毛细管电泳-飞行时间质谱法(time of flight mass spectrometry, TOF MS)进行尿蛋白质组学分析,得到由50个肽组成的早期COVID-19感染的尿蛋白质组学生物标志物COV50,可以用于预测SARS-CoV-2的死亡和疾病进展。Trung等^[9]^在*ACS Central Science*报道了利用毛细管电泳-三重四极杆质谱(capillary electrophoresis-tandem mass spectrometry, CE-MS/MS)联用技术结合稳定同位素^13^C追踪揭示复杂的依赖多肌醇多磷酸磷酸酶1(multiple inositol polyphosphate phosphatase 1)的肌醇磷酸盐网络方法,结果表明稳定同位素代谢标记结合核磁共振波谱和CE-MS/MS构成了注释各种生物环境下磷酸肌醇网络的强效工具。

已报道方法的新应用 Islam等^[10]^在*Microbiome*阐明了影响粪便微生物群移植(fecal microbiota transplantation, FMT)治疗效果的潜在因素,直接使用了Sugiura等^[11]^2011年在*PLoS One*上发表的CE-MS法,完成了粪便的代谢组学分析,为成功预测FMT潜在标记物提供了数据。值得关注的是,Sugiura等是在参考其他方法的基础上,同时建立了阳离子代谢和阴离子代谢的CE-MS分析方法。其中,阳离子代谢产物分析方法是改进了Soga等^[12]^2000年发表在*Analytical Chemistry*的CE-MS分析氨基酸的方法,阴离子代谢产物分析方法是改进了Soga等^[13]^2007年发表在*Journal of Chromatography A*的压力辅助CE-MS分析核苷酸的方法。

### 1.2 重要综述

*TrAC-Trends in Analytical Chemistry*发表了6篇综述。

CE技术 Wu等^[14]^介绍了cIEF-WCID技术的特点及其在生物制药产品的配方和稳定性研究、质量控制、蛋白质的糖基化、工艺优化研究、蛋白质电荷异质性表征方面的应用,展望了成像cIEF与荧光检测、免疫分析、MS、微芯片的联用技术及其在监测蛋白质相互作用过程中的应用前景。Liu等^[15]^认为cIEF、CE-MS和毛细管电泳-十二烷基硫酸钠(capillary electrophoresis-sodiumdodecyl sulfate, CE-SDS)可用于体外药物抗体比(drug-to-antibody ratio)的表征,其中CE-SDS的精密度虽然不高,但可以作为其他平台的补充验证方法使用。Quintas等^[16]^认为手性离子液体、深共晶溶剂(deep eutectic solvents, DESs)和表面活性剂等作为手性选择溶剂的替代溶剂,能够提高CE分离对映体的分辨率。Kaljurand等^[17]^综述了便携式和现场CE仪器、开放访问CE仪器、便携式CE仪器运行中遇到的问题及解决方法,充分体现了CE作为绿色分析技术的潜能。

CE-MS技术 Zhang等^[18]^综述了毛细管电泳-非变性质谱技术(capillary electrophoresis-native mass spectrometry, CE-nMS)在探测蛋白质高阶结构(higher-order structures)方面的最新进展,介绍了CE-nMS分离和测定不同蛋白质形态的工作原理及其相关应用,详述了所在课题组基于CE-nMS开发的测定蛋白质高阶结构相关参数的新方法及其在蛋白质流体动力学半径、有效电荷、三维椭球形状和蛋白质-配体结合常数快速测定中的应用。Della Posta等^[19]^综述了以CE为代表的电迁移技术与MS联用技术的最新进展,包括CE工作原理和仪器、CE与质谱的接口、二维分离系统与MS联用及其选择性应用等。

其他相关综述 Shah等^[20]^在*Journal of Pharmaceutical Analysis*上概述了近十年来CE技术在药物杂质分析中的应用研究进展,以药物化合物为例强调了CE的优势,可以帮助科学家、其他研究人员和学生了解CE技术在药物杂质分析中的应用。Meng等^[21]^在*Trends in Food Science & Technology*上综述了蜡样芽孢杆菌及蜡样毒素的研究进展,介绍了胶束电动毛细管色谱法(micellar electrokinetic capillary chromatography, MEKC)在检测稻米样品中的蜡样毒素和评价芽孢杆菌的蜡液产量中的应用,解释了该法简单、高效但使用受限的原因是检出限比MS低1到2个数量级。

## 2 10.0≥IF≥5.0的期刊

### 2.1 *Analytical Chemistry*

#### 2.1.1 CE的新方法和新应用

CE新方法 Vaghef-Koodehi等^[22]^提出了用于微米级微粒高分辨率分离的CE方法,基于绝缘子的电动化系统可通过改变电势大小将线性和非线性电动力学结合,实现高效的微粒分离,有望用于高度相似、电荷差更小的微米级颗粒的分离。Gao等^[23]^开发了非对称流场流分离(asymmetrical flow field-flow fractionation)和CE的离线耦合技术,可快速定量生物样品中的细胞外囊泡(extracellular vesicles, EVs),大大降低基质干扰,对研究细胞产生的EVs和EVs亚群或临床样品中存在的EVs具有应用价值。Gallion等^[24]^以原代人结肠上皮细胞为模式细胞,建立了毛细管电泳-荧光检测法(capillary electrophoresis with fluorescence detection, CE-F)测定单细胞酶活性的新方法,有望解决细胞酶底物产生的荧光信号对待测物的干扰问题。Lavigne等^[25]^建立了毛细管电泳间接紫外吸收法定量分析纳升级样品的方法,可在没有参考物质的情况下对有机分子和离子进行定量分析。Casto-Boggess等^[26]^建立了自动纳米凝胶毛细管电泳法,不需要将酶与抑制剂预混合,可减少酶的消耗,适用于多种不同抑制剂的筛选。

用于筛选核酸适配体 Teclemichael等^[27]^利用亲和力测定证明了在核酸适配体筛选过程中,非结合库的分数(fraction of the unbound library, *R*)用于批量亲和力计量理论上优于平衡解离常数(equilibrium dissociation constant, *K*_d_),提出了解决*R*在实际测定中过于依赖目标浓度的新方法,避免了在每一轮实验中反复测定确认浓度,适用于不同核酸适配体的筛选工作。Le等^[28]^用CE定量表征蛋白质-DNA复合物在核酸适配体筛选中与非复合物的分离,评估了平衡混合物的非平衡毛细管电泳(nonequilibrium capillary electrophoresis of equilibrium mixtures, NECEEM)和理想过滤式毛细管电泳(ideal-filter capillary electrophoresis, IFCE),发现IFCE更适合相对分子质量大的蛋白质靶标,而NECEEM更适于小尺寸蛋白质靶标。Nagano等^[29]^借助微珠辅助CE(microbead-assisted capillary electrophoresis)筛选技术,构建了基于寡核苷酸检测甲氨蝶呤(methotrexate)的系统,检测成本低,准确性高。

用于抗体药物分析 Malburet等^[30]^建立了表征用作mRNA疫苗传递载体的脂质纳米颗粒(lipid nanoparticles, LNPs)的平均尺寸、尺寸分布以及电荷密度的新方法,其中使用CE测定了LNPs的总电荷。McClain等^[31]^利用免疫球蛋白G降解酶(immunoglubulin G-degrading enzyme of streptococcus pyogenes)产生F(ab')2和Fc片段,从亲本种中分离出被剪切的亚基种,提高了目标物的纯度,解决了毛细管凝胶电泳法(capillary gel electrophoresis, CGE)分离非典型片段时分辨率低的问题。Filep等^[32]^以硼酸交联葡聚糖为筛分基质,利用CGE研究了治疗性单克隆抗体奥马珠单抗(omalizumab)轻链和重链亚基峰的电迁移扩散,引入硼酸盐梯度介导瞬时迁移率匹配的方法,在一次运行中同时进行轻链和重链峰对称性的迁移率匹配,为实现毛细管孔径梯度凝胶电泳提供了可能的解决方案。

其他 Dvořák等^[33]^搭建了自动顺序注射(automated sequential injection)和CE全自动分析干血斑(dried blood spots, DBSs)的联用平台,提供了速度快、灵活度高的DBSs洗脱和分析过程,用于以生物材料为代表的广泛分析。Wang等^[34]^证明了扩散系数接近的物质也存在瞬态不完全分离状态,通过检测由大小相似分子组成的复合物的*K*_d_值得到瞬时不完全分离精确常数,这可以作为表征蛋白质-适配体在溶液中的结合亲和力的方法。Torres等^[35]^研究发现了乙胺作为DESs可以形成非极性、极性和不带电D/L型氨基酸荧光标记反应的溶剂,利用CE-LIF对含有DESs基质样品中的氨基酸进行了检测。此外,Kehl等^[36]^研制了高压兼容流量传感器,以nL/min的分辨率监测分离过程中毛细管内的体积流量,以信号对电迁移速度绘制电泳图可以矫正迁移时间。Hong等^[37]^详细讨论了构建基于细胞、细菌和病毒的小型化系统的各种策略,介绍了提高微尺度系统性能的新方法以及将生物组件引入微尺度系统的优点。

#### 2.1.2 CE-MS新应用和新硬件

蛋白质多肽分析 Xu等^[38]^提出了自动非变性毛细管等电聚焦质谱(nondenaturing capillary isoelectric focusing-mass spectrometry)揭示完整蛋白质复合物微观异质性的方法,比nCE-MS的分辨率更高。DeLaney等^[39]^将CE-ESI MS与TOF MS串联使用,对小鼠脑血管紧张素肽进行微量分析,检出限可达10^-13^ mol或18000个拷贝数水平,可广泛用于蛋白质与多肽分析。

蛋白质组学 Johnson等^[40]^构建了HeLa细胞小型化的溶液内消化工作流程,开发了超灵敏的CE-MS/MS方法,用于有限生物样本自下而上的蛋白质组学分析,总蛋白含量降低至0.1 ng,优于目前报道的基于CE-MS/MS的蛋白质组学分析方法。Salim等^[41]^建立了在线适配体亲和固相萃取和固定化酶微反应器毛细管电泳-质谱联用全集成的方法(aptamer affinity solid-phase extraction and immobilized enzyme microreactor capillary electrophoresis-mass spectrometry, AA-SPE-IMER-CE-MS),用于敏感靶向蛋白质自下而上分析的无阀分析(valve-free method),能够从小于100 μL的样品中清理和预浓缩高达100倍的目标蛋白质。Johnson等^[42]^针对有限的样品,取消了样品处理和转移步骤,直接在分离毛细管中处理完整的细胞,利用CE-MS/MS在线对小于10个的哺乳动物癌细胞和单个细胞分别进行自上而下的蛋白质组学分析,实验证明CE-MS/MS与毛细管在线样品处理在单个细胞和有限样品的高灵敏度分析中具有巨大潜力。

增敏技术 Kok等^[43]^评估了*N*-(4-氨基甲基苯基)吡啶盐(*N*-(4-aminomethylphenyl)pyridinium)、2-甲基吡啶胺(2-picolylamine)和*N*,*N*-二乙基乙二胺(*N*,*N*-diethylethylenediamine)与脂肪酸的衍生化效果,验证了CE-MS在多种脂肪酸的高效分离和灵敏检测方面有巨大的发展潜力。Wagt等^[44]^使用静态涂覆的中性毛细管减少电渗流(electro-osmotic flow, EOF),建立了零流(zero flow)CE-MS技术,可对多种糖类异构体进行分离,实现了*N*-聚糖异构体高灵敏度的结构表征。Tan等^[45]^采用高压方波代替传统迁移毛细管电泳法中的直流高压作为分离电压,加强了质谱信号强度,降低了背景干扰,促使金属阳离子与核酸阴离子的解离,获得最高可达10倍的更清晰的质谱图和更强的核酸离子峰。

硬件研发 Elshamy等^[46]^设计了新的纳米流鞘,用于振动锐缘喷雾电离(vibrating sharp-edge spray ionization, VSSI)中,降低了分离毛细管容积流量的影响,减少了CE-MS联用过程中对分离流速的依赖,促进目标物转移到MS,提升了纳米流鞘VSSI喷雾电离的功能,增加了分离条件的灵活性。Song等^[47]^建立了表征单细胞内极性代谢物的特异性分析方法,使用探针电泳质谱(probe electrophoresis mass spectrometry)对单细胞代谢物进行电泳采样,并结合纳米ESI-MS进行了单细胞代谢物的特异性分析。

### 2.2 *Analytica Chimica Acta*

#### 2.2.1 CE新方法和新应用

Tang等^[48]^将循环酶催化信号放大技术(cyclic enzymatic signal amplification)与CE-UV相结合,建立了无标记的C反应蛋白(C-reactive protein)检测方法,在人血清的体积分数为1%的反应体系中,检出限为4 ng/mL,重复性好、可靠性高。Horká等^[49]^利用电泳法、瞬间等速电泳法(transient isotachophoresi, tITP)和MEKC法,在60 min内鉴定了不同基因型的耐甲氧西林金黄色葡萄球菌(methicillin-resistant Staphylococcus aureus, MRSA),还能部分分离单个基因型组内的MRSA分离株。Duša等^[50]^使用cIEF对113种新型低分子质量等电点标记物进行高度可重复的分析,确定了其精确pI值,在pH 2.5~11内完成了pH梯度覆盖。Li等^[51]^使用疏水DESs(HDESs)左旋薄荷醇和辛酸作为假固定相(pseudo-stationary phase, PSP)建立了基于DESs的毛细管电泳法,并证明了HDESs+羟丙基-*γ*-环糊精双PSP系统可以很好地分离13种芳香酸组成的复杂混合物。

#### 2.2.2 CE-MS新方法和新应用

Malá等^[52]^首次报道了用于阳离子电解质系统的设计,结合CE-ESI MS对水中三嗪类除草剂阿特拉津(atrazine)和特丁津(terbuthylazine)的降解产物进行分析,检出限可达到(0.5~1)×10^-9^mol/L。López-López等^[53]^优化了聚乙烯醇涂覆毛细管的条件,为CE-MS用于阴离子分析提供了可靠且灵敏的方法。Štěpánová等^[54]^从样品的制备、蛋白质吸附的抑制、电渗流的控制、用特定的CE分离蛋白质以及用各种检测技术进行蛋白质鉴定和定量等方面,详细综述了CE在2017~2021年间分离和分析蛋白质的相关进展。此外,CE在药物-蛋白质相互作用机理^[55]^、单抗的糖基化多样性^[56]^和核酸适配体的筛选^[57]^中也有应用。

### 2.3 *Talanta*

#### 2.3.1 涂层CE法

T $\dot{u}$ ma等^[58]^使用聚(丙烯酰胺共钠2-丙烯酰胺2-甲基丙磺酸钠)阴离子共聚物(poly acrylamide-co-sodium-2-acrylamido-2-methylpropanesulphonate)涂层毛细管法,从人血清中分离作为人体生酮指示剂的3-羟基丁酸酯(3-hydroxybutyrate),检出限为0.43 ng/mL。Pan等^[59]^使用荧光标记的通用引物结合基因特异性引物和两个通用的非人类序列,使用安捷伦(Agilent)公司的DB-17型毛细管建立了快速分析人类红色和绿色色素基因外显子1~5的CE方法。Li等^[60]^基于自制聚乙烯醇涂层和Guarant品牌涂层毛细管,使用8-氨基芘-1,3,6-三磺酸盐(8-aminopyrene-1,3,6-trisulfonate)、Teal和Turquoise牌染料,利用CE-LIF对N-聚糖进行了交叉检验,改进了用于精确识别N-聚糖结构的多路复用CE方法。

#### 2.3.2 其他

Horká等^[61]^采用聚合体增强瞬间等速电泳和MEKC中带电细菌细胞扫描相结合的方法,分离了20株临床痤疮丙酸杆菌(*Cutibacterium acnes*, *C. acnes*)分离株,在20 min内完成了个体系统类型的正确分类,所有被检细菌的检出限为5×10^5^ 细胞/mL(100 nL注射样品中有50个细胞)。Zhang等^[62]^利用3D打印技术制作固定光学元件的积木,形成光源、透镜、毛细管、二向色镜和荧光探测器模块并结合市场上的乐高积木,开发了模块化组装的CE-LIFGO系统,将其应用于DNA片段的分析,证明了其在生化分析中应用的可能性。此外,CE在单细胞药物反应测量技术^[63]^和溶血磷脂酸检测^[64]^中也有应用。

### 2.4 *Food Chemistry*

Horká等^[65]^将tITP和MEKC结合,实现了大体积样品牛奶中蛋白质的在线富集。将tITP/MEKC与cIEF相结合,实现了脱脂牛奶中蛋白质的在线浓缩、分离、鉴定,该方法对*α*-乳白蛋白(*α*-lactalbumin)和*β*-乳球蛋白(*β*-lactoglobulin, *β*-LG)的遗传变种(*β*-LG A和*β*-LG B)的检出限分别为1.2×10^-3^ ng/L、1.0×10^-3^ ng/L和1.0×10^-3^ ng/L。此外,在啤酒花中*α*和*β*酸的分析^[66]^、天然碳水化合物的样品制备和精细化学结构的测定^[67]^的综述中也有CE相关内容。

## 3 IF<5.0的重要期刊

### 3.1 *Journal of Chromatography A*

#### 3.1.1 与扫集技术联用提升灵敏度

Cheng等^[68]^建立了场放大进样(field amplified sample injection)、扫集(sweeping)和胶束塌陷聚焦(analyte focusing by micelle collapse)三步叠加法,测定了卷烟产品中烟草特有的5种致癌亚硝胺的含量,利用化学计量学辅助设计筛选最佳电泳条件,5种目标物的检出限均达到ng/g级。Chu等^[69]^将离线盐析辅助液-液萃取与在线三步CE堆叠法结合使用,检测复杂生物样品中微量抗肿瘤吲哚类生物碱,检出限为ng/mL,可作为测定复杂样品基质中痕量吲哚类生物碱的参考方法。Semail等^[70]^建立了双叠tITP结合扫集胶束电动色谱-二极管阵列检测器在环境水样中测定非甾体抗炎药的方法,检出限达到ng/mL。Zhou等^[71]^建立了阳离子选择性耗尽进样-扫集-环糊精修饰电动色谱拆分西酞普兰(citalopram)对映体的方法,并用于草酸艾司西酞普兰(escitalopram oxalate)的检测及西酞普兰对映体的立体选择性药代动力学研究,样品消耗低,灵敏度高,可靠性好。

#### 3.1.2 表面活性剂辅助提升分辨率

Yu等^[72]^以3-(*N*,*N*-二甲基棕榈酰亚胺)丙烷磺酸盐(3-(*N*,*N*-dimethylpalmitylammonio)propane sulfonate)为双离子表面活性剂建立了电动进样和MSS(micelle-to-solvent stacking)两步堆叠法,用于食品中4种砷(As)形态的分离浓缩,即As(Ⅴ)、As(Ⅲ)、砷酸盐六水甲醇二钠(disodium methyl arsonate hexahydrate, MMA)和二甲胂酸(dimethylarsinic acid),富集因子达840~3820,检出限为0.382~0.911 ng/mL。Yan等^[73]^采用超声强化和Brij-35表面活性剂辅助的分散液-液微萃取与聚二烯丙基二甲基胺盐酸盐(poly(diallyldimethylammonium chloride))介导的CE叠加技术,建立了简单、快速、灵敏的测定人体液中米氮平(mirtazapine)及其代谢产物对映体的方法,为利用双富集CE体系检测手性化合物提供了有效的参考方法。

#### 3.1.3 CE-MS新方法

Carbonell-Rozas等^[74]^提出了MEKC-MS法,结合QuEChERS前处理技术用于测定花粉和蜂蜜样品中的新烟碱(neonicotinoids)和杀菌剂啶酰茵胺(boscalid),检出限分别为3.5 ng/g和4.0 ng/g。Ma等^[75]^合成了具有永久正电荷的新型聚糖标记试剂(4-肼基丁基)三苯基溴化膦((4-hydrazidebutyl)triphenylphosphonium bromide),用于基质辅助激光解吸电离(matrix-assisted laser desorption ionization, MALDI)质谱和ESI质谱等常见的生物质谱技术,可以提高MS检测低聚糖的信号强度,对麦芽糖糊精(maltodextrin)的检测信号强度可达10^6^。Hamidli等^[76]^筛选了静态线性聚丙烯酰胺修饰毛细管的最佳操作条件,建立了毛细管区带电泳(CZE)-MS测定人胰岛素及其6个类似物的方法,为具有相似或相同分子质量的胰岛素提供了结构信息和明确识别。Li等^[77]^证明了贝伐单抗(bevacizumab)的剪切变体可以通过CZE-MS和cIEF-MS方法直接识别,为传统的CE-SDS分析工作流程提供了强大的补充;CE-SDS、CZE-MS和cIEF-MS之间的交叉验证,使单克隆抗体大小变异分析更加全面。

### 3.2 *Electrophoresis*

#### 3.2.1 MEKC模式

Yao等^[78]^基于反相MEKC法建立了以2,2'-双(3-乙基苯并噻唑啉-6-磺酸)铵盐(2,2'-bis(3-ethylbenzothiazoline-6-sulfonic acid) ammonium salt)为氧化自由基在线评价三叶糖脂清片(sanyetangzhiqing, SYTZQ)总抗氧化活性的方法,准确计算了SYTZQ的总抗氧化活性,为药物抗氧化活性的评价提供了参考方法。Čokrtová等^[79]^建立了非水MEKC分析合成乳酸基液晶纯度的方法,用溴化十六烷基三甲铵作表面活性剂,获得了更好的迁移时间重复性,补充了高效液相色谱法和超临界流体色谱法的结果,降低了检测成本。

#### 3.2.2 其他

Candreva等^[80]^利用成像cIEF紫外和荧光双检测分别对0.02~0.98 mg/mL内的mAbs进行电荷变化分析,可用于单克隆抗体组合产品的质量控制。此外,Huang等^[81]^开发了基于python的脚本,用于半靶向代谢组学研究双压力CE(dual pressure CE)的标记物选择、迁移时间校正和峰值比对,明显提高了样品代谢物的迁移时间精度,相对标准偏差优化到小于1.8%。

## 4 国内进展

### 4.1 新材料辅助提升CE灵敏度

李超等^[82]^基于场放大进样技术,开发了以硫掺杂的石墨烯量子点(S-GQDs)为载体的双重富集CE法,用于检测盐酸二甲双胍制剂中的三聚氰胺和双氰胺,检出限分别为2.6×10^-5^ mol/L和5.7×10^-5^ mol/L。杨钰婷等^[83]^利用比表面积高达2630 m^2^/g的石墨烯纳米材料制备了SPE小柱,建立了SPE-CE检测婴儿奶嘴样品中双酚A的方法,在0.05~80 μg/mL内表现出良好的线性关系,检出限低至0.02 μg/mL。谭凤玉等^[84]^以搅拌棒为载体的聚己内酰胺膜作为固相萃取剂,结合场放大进样在线富集技术和毛细管电泳非接触式电导法测定了果酱中胭脂红和苋菜红的含量,检出限分别为8.1 μg/L和8.2 μg/L。张雪娇等^[85]^以丝氨酸-纳米金为手性选择剂,采用CZE对异丙肾上腺素、肾上腺素和去甲肾上腺素药物进行手性拆分,分离度分别达到3.19、2.85和3.77。陈国宏等^[86]^以牛血清白蛋白和磁珠为模式反应物,建立了原位电泳洗脱模型,用于在荧光标记后快速去除多余的荧光探针,降低荧光背景响应,提升了检测灵敏度。

### 4.2 CE方法潜在应用范围的拓展

马菁菁等^[87]^将多重PCR毛细管电泳相结合,建立了针对支原体、金黄色葡萄球菌、肺炎克雷伯菌、铜绿假单胞菌、阴沟杆菌、白色假丝酵母菌和鲍曼不动杆菌的快速检测体系,可在48 h内完成检测,有望在常见儿童呼吸道病原体临床检测上大规模推广应用。贺木易等^[88]^采用外标Hummel-Dreyer(HD)CE法研究了双酚E与血清白蛋白的相互作用,为体外环境的双酚类化合物毒理效应研究提供了有益参考。蔡先洪等^[89]^基于CE与指数富集配体系统进化(systematic evolution of ligands by exponential enrichment, SELEX)技术筛选了维吉霉素M1(virginiamycin M1)的核酸适配体,为其高效快速检测提供了技术支持。梁子其等^[90]^研发了多通道电容耦合式非接触电导检测装置并开发了自动测量软件,解决了自由流电泳和往复式自由流等电聚焦电泳的在线检测问题,拓展了CE技术的应用范围。

此外,王文波等^[91]^依据国际人用药品注册技术协调会(ICH)Q2_R1指导原则和2015年版《中华人民共和国药典》通则9101,组织开展了基于CE-LIF检测单抗N-糖谱的方法学联合验证,结果表明CE-LIF可用于单抗N-糖谱检测。迟忠美等^[92]^综述了CE-MS在手性化合物分析领域的技术、手性选择剂的使用以及在医药等领域应用方面的研究进展,讨论了不同手性分析模式的局限性,为未来CE-MS在手性分离分析技术方面的发展及应用提供了借鉴。

## 5 结论

纵观2022年全年,国际方面,CE依旧在临床研究、医用蛋白质和药物分析方面发挥优势,新材料和富集技术与MEKC模式的联用最为突出,CE-MS尤其是硬件方面的报道相较2021年有所下降;发表时间较早或发表在IF较低期刊上的方法,在新领域的应用结果也可以发表在IF较高的期刊上,为CE的应用提供了新思路。国内方面,CE技术在临床、卫生和食品安全等领域有了更多的应用报道。

以上内容难免有遗漏和不妥之处,请广大学界同仁及应用从业人员批评指正。
